# Neuroanatomical substrates for the volitional regulation of heart rate

**DOI:** 10.3389/fpsyg.2015.00300

**Published:** 2015-03-25

**Authors:** Catherine L. Jones, Ludovico Minati, Yoko Nagai, Nick Medford, Neil A. Harrison, Marcus Gray, Jamie Ward, Hugo D. Critchley

**Affiliations:** ^1^Clinical Imaging Sciences Centre, Brighton and Sussex Medical School, University of SussexBrighton, UK; ^2^Department of Psychiatry and Sackler Centre for Consciousness Science, Clinical Imaging Sciences Centre, University of SussexBrighton, UK; ^3^IRCCS Istituto Neurologico Carlo BestaMilano, Italy; ^4^Sackler Centre for Consciousness Science, University of SussexBrighton, UK; ^5^Sussex Partnership NHS Foundation TrustWorthing, UK; ^6^Gehrmann Laboratory, University of Queensland, BrisbaneQLD, Australia; ^7^School of Psychology, University of SussexBrighton, UK

**Keywords:** autonomic, biofeedback, brain imaging, emotion, heart rate, interoception

## Abstract

The control of physiological arousal can assist in the regulation of emotional state. A subset cortical and subcortical brain regions are implicated in autonomic control of bodily arousal during emotional behaviors. Here, we combined human functional neuroimaging with autonomic monitoring to identify neural mechanisms that support the volitional regulation of heart rate, a process that may be assisted by visual feedback. During functional magnetic resonance imaging (fMRI), 15 healthy adults performed an experimental task in which they were prompted voluntarily to increase or decrease cardiovascular arousal (heart rate) during true, false, or absent visual feedback. Participants achieved appropriate changes in heart rate, without significant modulation of respiratory rate, and were overall not influenced by the presence of visual feedback. Increased activity in right amygdala, striatum and brainstem occurred when participants attempted to increase heart rate. In contrast, activation of ventrolateral prefrontal and parietal cortices occurred when attempting to decrease heart rate. Biofeedback enhanced activity within occipito-temporal cortices, but there was no significant interaction with task conditions. Activity in regions including pregenual anterior cingulate and ventral striatum reflected the magnitude of successful task performance, which was negatively related to subclinical anxiety symptoms. Measured changes in respiration correlated with posterior insula activation and heart rate, at a more lenient threshold, change correlated with insula, caudate, and midbrain activity. Our findings highlight a set of brain regions, notably ventrolateral prefrontal cortex, supporting volitional control of cardiovascular arousal. These data are relevant to understanding neural substrates supporting interaction between intentional and interoceptive states related to anxiety, with implications for biofeedback interventions, e.g., real-time fMRI, that target emotional regulation.

## Introduction

States of physiological bodily arousal, including increased heart rate, are integral to the expression of negative emotions, including anxiety, and feed back to intensify affective feelings. Interventions that specifically target physiological arousal can diminish anxiety symptoms and emotional reactivity (Bonn et al., 1972). Physiological relaxation techniques, with or without biofeedback, contribute to strategies for anxiety management, often alongside cognitive behavioral therapy (Borkovec and Costello, 1993). Further, the intentional regulation of emotional states engages brain regions implicated in the control of peripheral as well as central arousal (Buhle et al., 2014). However, physiological arousal itself also accompanies non-emotional behavioral states, notably physical activity, which do not typically evoke negative feelings. One explanation for this discrepancy lies in the predictability and sense of control of internal physiological state mediated by the autonomic nervous system (Paulus and Stein, 2006; Seth, 2013).

Autonomic responses are integrated with emotional and motivational behaviors. Correspondingly, brain regions controlling behavior also directly or indirectly influence internal bodily arousal states via the autonomic nervous system. These bodily changes are themselves linked to activation within discrete brain regions. For example, stimulation of the human insula can evoke visceromotor changes (Penfield and Faulk, 1955; Oppenheimer et al., 1992) and insula damage may result in autonomic dysregulation (Oppenheimer et al., 1992; Meyer et al., 2004; Jones et al., 2010). Neuroimaging evidence also implicates regions including insula, anterior cingulate, and amygdala in interoception (sensing and representing the physiological state of the body) and accompanying feelings states (Damasio, 1994; Craig, 2002, 2009; Critchley et al., 2004; Harrison et al., 2010).

These same set of brain regions contribute to brain networks that are also implicated in executive, cognitive, and social functioning (Seeley et al., 2007; Sridharan et al., 2008; Limongi et al., 2014). Connectivity within such networks appear dynamically related to changes in peripheral cardiovascular state: thus, during the resting state, increases in heart rate variability fluctuate with increases in connectivity from dorsal anterior cingulate and amygdala to other cortical (cingulate insula and dorsolateral prefrontal cortex) and subcortical (basal ganglia and midbrain) centers (Chang et al., 2013). Cardiorespiratory effects similarly, contribute to connectivity strength within the ‘default mode’ network (encompassing medial prefrontal/rostral cingulate and medial parietal lobe). Removal of variance from physiological bodily responses diminishes experimental sensitivity to task-related changes in brain activity (van Buuren et al., 2009). Nevertheless, these passive relationships raise important questions regarding the functional impact of such heart-brain interactions.

The neural mechanisms supporting this link between peripheral arousal and emotional feelings have attracted therapeutic attention. Biofeedback of brain activity (neurofeedback) has been explored in this context: here, the immediate explicit (visual) presentation of changes in neural activation or connectivity can be used as a ‘training signal’ that enables a participant to learn to wilfully modulate neural responses to affect associated psychophysiological processes. For example, interventions that target insular cortex (or connected brain regions) have been explored in the management of affective symptoms and chronic pain disorders. Anterior insula in particular, has been the target of neurofeedback studies using real-time functional magnetic resonance imaging (fMRI; e.g., Caria et al., 2007; Frank et al., 2012). Autonomic biofeedback tasks (using peripheral response) can also be used to extend knowledge about neural substrates supporting the functional integration of cognition and internal bodily states of arousal: the proposed role of anterior insula as the substrate for (emotional) feeling states arising from internal visceral states (Craig, 2002, 2009; Critchley and Harrison, 2013), predicts that this region is likely to be involved in the volitional/intentional regulation of physiological state. Similar arguments apply also to closely connected regions such as anterior cingulate cortex, which is implicated in both emotional autonomic arousal and emotion awareness (Lane et al., 1998). In fact, anterior cingulate cortex is observed to be activated during performance of electrodermal biofeedback tasks (Critchley et al., 2001, 2002a; Nagai et al., 2004a).

In the present paper, we focused on the control of heart rate. At rest, heart rate modulation is achieved through changing the balance between both sympathetic and parasympathetic drive, hence it is closely related to baroreflex mechanisms that underlie heart rate variability. We chose to focus on identifying regional brain centers contributing to the active/intentional regulation of heart rate (arguably, a more intuitively accessible physiological response than heart rate variability). We tested the notion that both sensing internal bodily states and regulating these states activate cortical regions such as insula, where ascending interoceptive representation appear to be integrated with conscious perception (Critchley et al., 2002b; Gianaros et al., 2012; Gray et al., 2012). We investigated the ability to wilfully modify heart rate, in the presence of biofeedback (visual feedback of their actual heart rate), no feedback, or false feedback. A key prediction was that the insula, alongside anterior cingulate cortex, and dorsal brainstem, would be engaged during biofeedback regulation of heart rate. Thus, we predicted that the presence and veracity of the feedback would modulate both behavior (successful performance of the task) and associated neural activity within brain regions supporting regulation and representation of autonomic bodily responses. Ultimately, we were motivated by a perceived relevance to emotional regulation and anxiety (e.g., Clark, 1986; Beck and Clark, 1997; Paulus and Stein, 2006; Dunn et al., 2010; Domschke et al., 2011). Hence participants also completed an anxiety inventory to test the prediction that effective autonomic regulation (i.e., successfully increasing and decreasing heart rate) would be related to *reduced* levels of anxiety. To our knowledge, this is the first study to investigate the effect of feedback on the modulation of heart rate while using fMRI to map the neural representations. A further novel aspect is the exploration of the relationship between anxiety symptoms and the capacity for volitional autonomic regulation.

## Materials and Methods

### Participants

Fifteen right-handed, healthy participants (Five male), mean age 25 ± 10 years, were enrolled. All participants were screened to exclude neurological and psychiatric disorders. The study was approved by the Brighton and Sussex Medical School Research Governance and Ethics Committee. Each participant was fully informed and gave written consent to take part in this neuroimaging study entitled ‘Biofeedback control of heart rate.’

### Experimental Design

Participants knowingly engaged with an experimental task involving intentional modulation of heart rate. The experiment involved six task conditions within a 2 × 3 factorial design. One factor was the objective, i.e., direction of intended heart rate change, where participants were required to try to increase or decrease their heart rate as an index of cardiovascular arousal [‘arousal’/increase and ‘relaxation’/decrease. The use of terms arousal and relaxation to refer to these physiological/autonomic changes, associated with increased sympathetic and decreased parasympathetic effects, is well established within the literature (e.g., from our own laboratory Critchley et al., 2000a,b, 2001, 2002a)]. The second factor, presence and veracity of visual feedback had three levels (true feedback – which accurately reflected heart rate; false feedback – randomly fluctuating information and absent feedback – no feedback given). Each condition was performed twice by each participant and presented in pseudorandom sequences that avoided immediate repetition of the same task condition.

**FIGURE 1:**
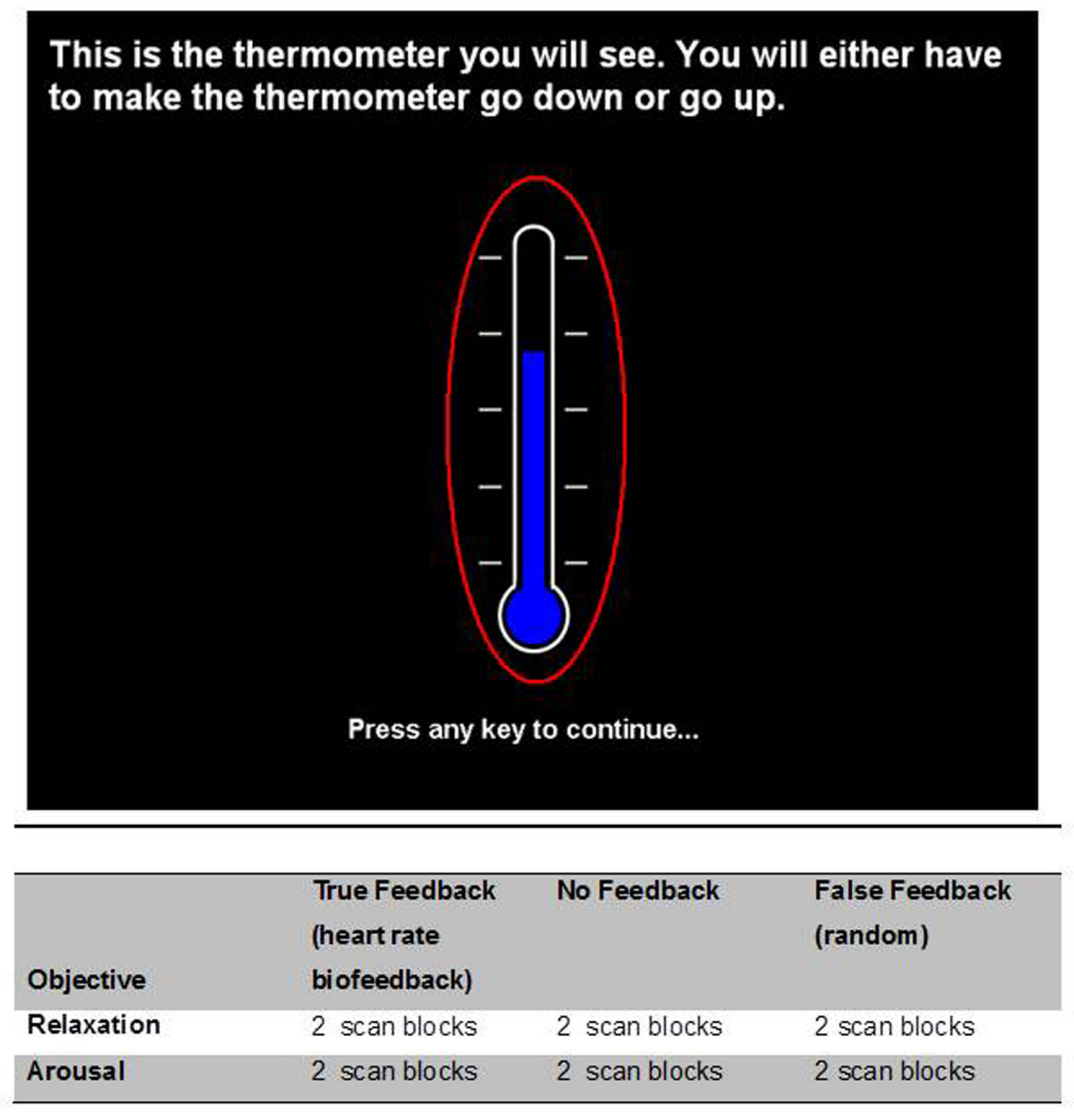
**Visual display of feedback ‘thermometer.’** There were six task conditions within the experiment which embodied a 2 × 3 factorial design. The factors were (1) the direction of intended heart rate change (increase = ‘arousal’/and decrease = ‘relaxation’) and (2) the presence and veracity of visual feedback (true feedback accurately reflecting heart rate; false feedback, i.e., random fluctuation and absent feedback). Each condition was performed twice by the participant and presented in pseudorandom sequences that avoided immediate repetition of the same task condition. Participants were instructed to use arousal to make the thermometer level rise in the increased heart rate conditions and relaxation in the lower in the heart rate conditions. Below are tabulated the biofeedback task conditions undertaken during the course of the experiment.

Heart rate and breathing rate were monitored continuously throughout the tasks. The biofeedback signal of physiological relaxation and arousal was represented by a visible thermometer with a blue bar that reflected heart rate (**Figure 1**). The starting point (near top or bottom) and approximate sensitivity was tailored for each participant by task condition. In the relaxation conditions, participants were instructed by a cue to try to make the bar go down by physiologically relaxing, conversely in the arousal task participants were instructed to make the bar rise by becoming more physiologically aroused. Thus the nebulous terms relaxation and arousal were operationalized, as in previous studies, to refer to heart rate decreases and increases, respectively. In the false feedback condition, the bar fluctuated following a smooth random walk. In the no-feedback condition there was no bar, with only the outline of a thermometer displayed. All participants knew that the purpose of the study was to control their own heart rate. Participants were informed that the thermometer reflected their heart rate, where a rise in heart rate was displayed as a rise in the level depicted on the thermometer which signaled an increase in physiological arousal. Similarly, participants were told that a drop in heart rate was displayed as a lowering of the level depicted on the thermometer which in turn signaled a reduction in physiological arousal, which we termed ‘relaxation.’ They were advised (when possible) to use the feedback and were also told that, during scanning, the feedback would be altered or removed for some trials, which might make it harder for them to achieve the required increase or decrease in arousal/heart rate. Practice trials were performed before scanning, in which participants were given only true heart rate feedback and were instructed to make to bar go down by relaxing and to make the bar rise by increasing the level of arousal, operationalized to mean heart rate, and reinforced by this practice session. These instructions were carried over to their performance within the scanner. Here again, the instruction to volitionally increase heart rate was displayed by the visual cue ‘AROUSAL’ and the instruction to volitionally decrease heart rate was displayed by the visual cue ‘RELAXATION’ at the start of each 90 s block (replacing the text shown in the upper part of **Figure 1**). The false feedback conditions were not explicitly distinguished from the true feedback conditions.

Participants were naïve to the biofeedback exercise before the day of the experiment (i.e., in contrast to previous biofeedback tasks they were not over-trained). In the instructions, Participants were explicitly instructed not to close their eyes (a natural behavior when trying to relax) and told to try and maintain a constant breathing rate. Task conditions alternated between relaxation and arousal conditions with feedback blocks presented in a pseudorandom manner. Each block lasted for 90 s (**Figure 1**).

### Physiological Monitoring

**FIGURE 2:**
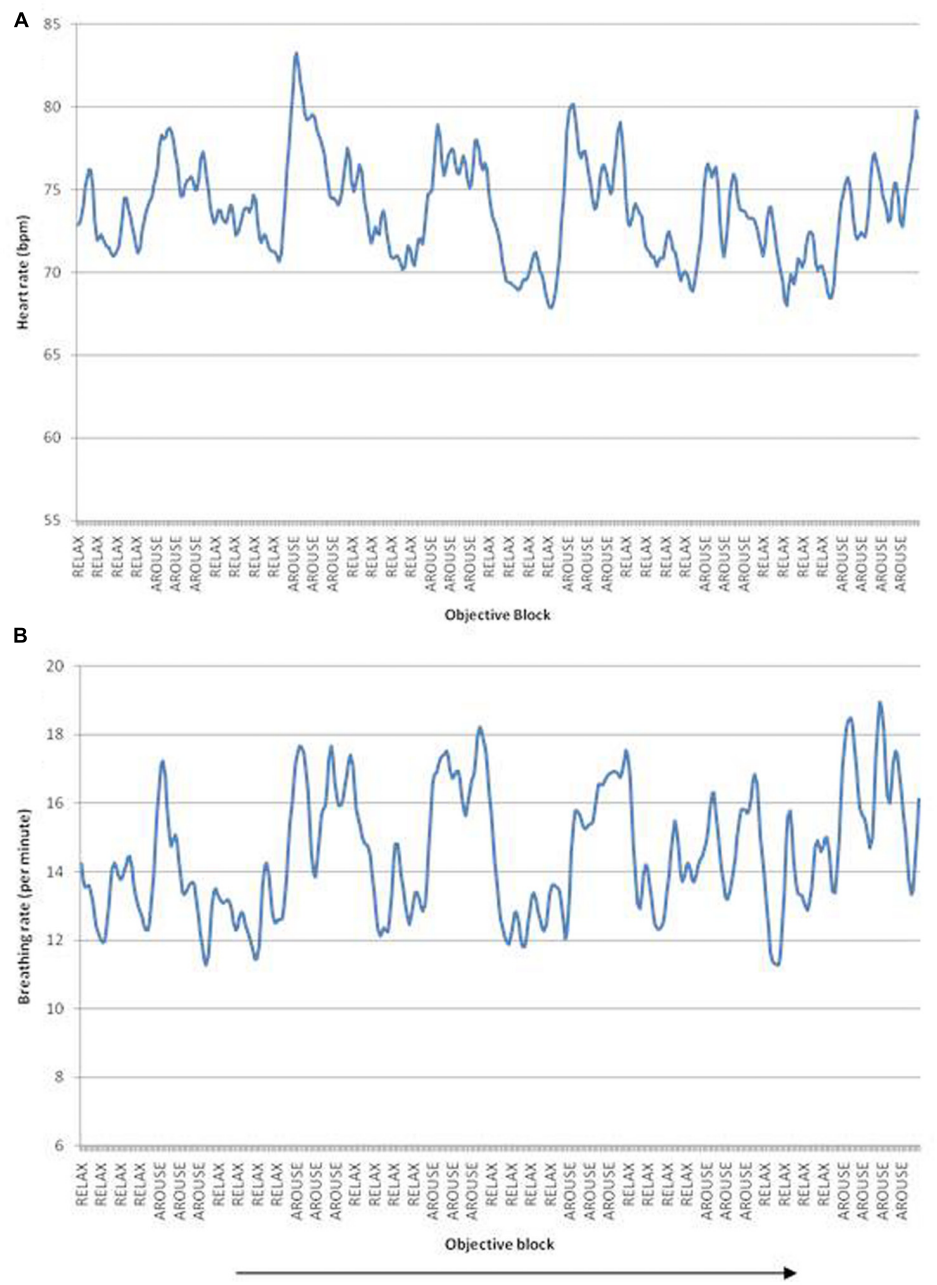
**(A)** Heart rate fluctuations over the scanning duration, peaks correspond to increased heart rate/arousal blocks, troughs correspond to decreased heart rate/relaxation blocks. **(B)** Breathing rate fluctuations over time. Arrow indicates passage of time. Data illustrated from a representative single participant.

Each participant was monitored during fMRI using pulse oximetry (Nonin 8600FO, Nonin Inc., Plymouth, MN, USA) with the sensor taped to the middle finger of the left hand. The fibreoptic cable was passed through the guide tube from the Faraday cage of the MRI scanner room to the control room where the analog outputs of the apparatus were fed via an A/D converter (CED1401) to a computer running Spike 2 Software (Cambridge Electronic Design, Cambridge, UK) and to a stimulus-control computer running Matlab (MathWorks, Nantick, MA, USA). The biofeedback components of the tasks (**Figure 1**) were run on this computer using Matlab scripts developed in-house: the signal was low-pass filtered at 1 Hz and processed with a peak-picking algorithm yielding beat-by-beat heart rate measurements. The resulting signal was epoched in the [-0.5,5] s peristimulus range and averaged across trials. Respiratory motor function was recorded within the MRI environment via respiratory bands, a technique referred to as remote pressure sensor respiratory plethysmography (Caldiroli and Minati, 2007). Again the signal was low-pass filtered at 1 Hz and processed with a peak-picking algorithm yielding breathing rate measurements (**Figure 2**).

### Behavioral Analysis

A 3 × 2 repeated measures ANOVA [feedback (True, None, False) × objective (Relaxation, Arousal)] was performed with heart rate as the dependent variable. A second ANOVA was conducted using breathing rate as a dependent variable. Each participant was debriefed to establish what particular strategies may have been used when performing the task. After debriefing participants also completed the Beck Anxiety Inventory (BAI, Beck et al., 1988). While none of the participants were above the clinical threshold for anxiety, scores were compared against performance on the biofeedback tasks.

### Neuroimaging Data Acquisition and Analysis

Each participant underwent neuroimaging on a Siemens Avanto 1.5 Tesla magnetic resonance imaging scanner. The participant was placed in the scanner with their head gently, yet firmly restrained within the head coil using vacuum cushions. During performance of the biofeedback tasks, T2^∗^-weighted echo planar data were acquired with near complete brain coverage (bi-commissural orientation for 21 slices, 5 mm thickness, no gap, TR = 2000 ms, TE = 50 ms, in-slice resolution 2 × 2 mm, matrix 80 × 128). A T1-weighted whole brain, high resolution structural scan was obtained at the end of the scanning study (magnetization-prepared rapid gradient-echo sequence, 0.9 mm isotropic voxels; TR = 1160 ms, TE = 4.44 ms, FoV 230 mm × 230 mm, matrix size 256 × 256, 50 slices) and used to co-register the functional dataset and screen for potential anatomical abnormalities.

Neuroimaging time-series datasets were analyzed as a block design using statistical parametric mapping (SPM8; Wellcome Trust Centre for Neuroimaging, UCL, UK) implemented in Matlab. Functional scans were realigned to correct for participant movements, slice-timing corrected, and co-registered with individual anatomy. Subsequently, all scans were transformed into MNI space. The scans were smoothed using an 8 mm full width at half maximum Gaussian filter. Two separate analyses were carried out: the first analysis tested for relationships between regional (blood oxygenation-level dependent, BOLD) activity and the different task conditions. The second analysis tested for regional activity sensitive to physiological fluctuation (heart rate and breathing rate) over the whole experiment.

In the first analysis, a design matrix modeled the six condition regressors for each participant, i.e., true_relaxation, true_arousal, none_relaxation, none_arousal, false_relaxation, false_arousal. There was no significant effect of the task conditions on head movement. Nevertheless, to improve sensitivity to neuraly mediated signal changes during the experiment, six movement regressors from the initial functional realignment were included in the design matrix as nuisance variables. The statistical maps were then entered into a second-level (i.e., group) random effects analysis, where a 2-way factorial analysis was employed to determine the presence of main effects and interactions between feedback and objective on regional activity at the population level. In the second analysis, an individual design matrix was created for each participant that included heart rate and breathing rate as regressors of interest. Again, movement parameters were included as nuisance regressors. These statistical maps were entered into second-level (i.e., group) analysis and one-sample *t*-tests were used to evaluate the significance of the effect of heart rate and breathing rate on regional neural (haemodynamic) response. In the neuroimaging results, activations which survive family-wise error (FWE) correction (*p* < 0.05) at the cluster level are reported, unless otherwise stated. Descriptions of anatomical location were determined using the anatomical toolbox for SPM (Eickhoff et al., 2005) and in addition the atlas of Duvernoy (1991).

**FIGURE 3:**
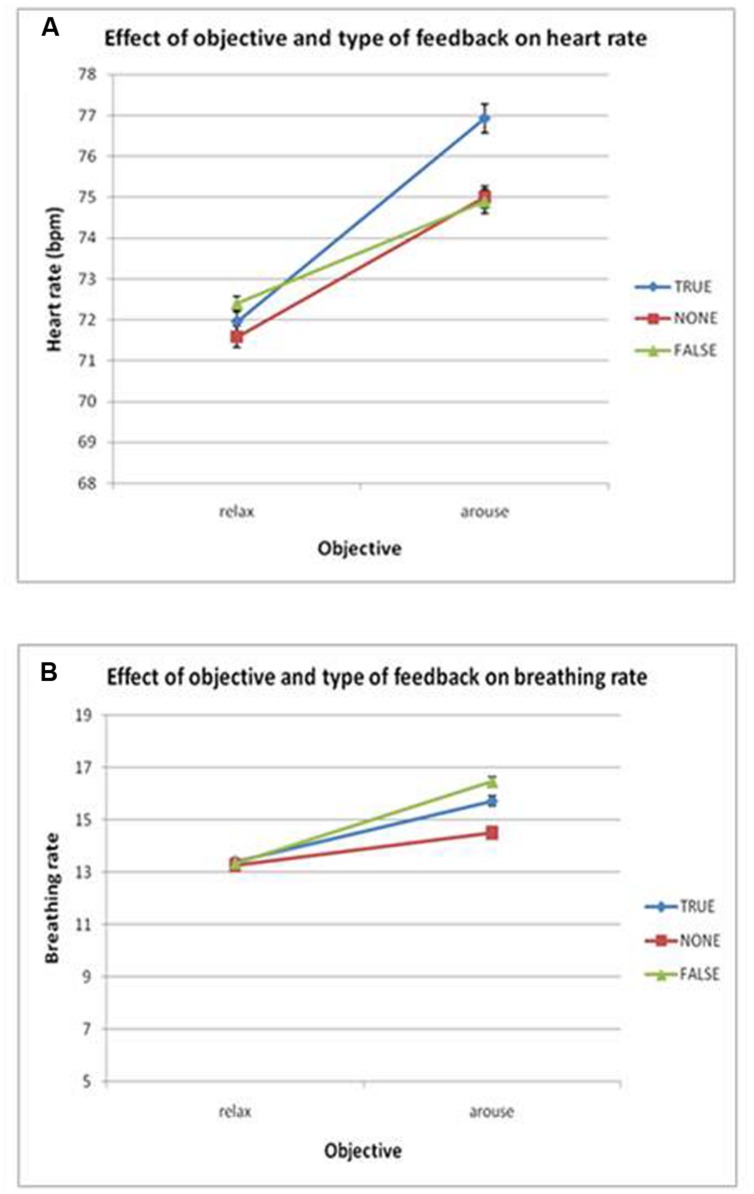
**(A)** Plots the group effects of objective and feedback on heart rate. There was a significant main effect of objective (*p* < 0.05) but no interaction of objective and feedback. **(B)** Breathing rate, no significant main effects or interactions were found. Error bars show SEM.

## Results

### Behavioral Results

Heart rate changed in accordance with the task instructions: across participants, heart rate averaged 76 bpm for the intended arousal condition compared to 72 bpm for the intended relaxation blocks. Thus task objective had a significant effect on heart rate, *F*(2,14) = 19.2, *p* < 0.01, η^2^ = 0.58, with (see **Figures 2 and 3A**). Surprisingly, however, there was no suprathreshold main effect of feedback type on heart rate across participants and no overall interaction between objective and feedback on heart rate. This suggests that as a group, participants were able to increase or decrease their heart rate according to the objective, but the presence of feedback did not significantly impact performance. There was a trend for heart rate to increase more in the accurate biofeedback condition during the intended arousal conditions (**Figures 2A and 3A**).

Using breathing rate as the dependent variable, we observed no significant main effects or interactions of objective and/or feedback on breathing rate, indicating that overall participants were able to modulate heart rate without significantly changing their breathing rate (see **Figures 2B and 3B**).

**FIGURE 4:**
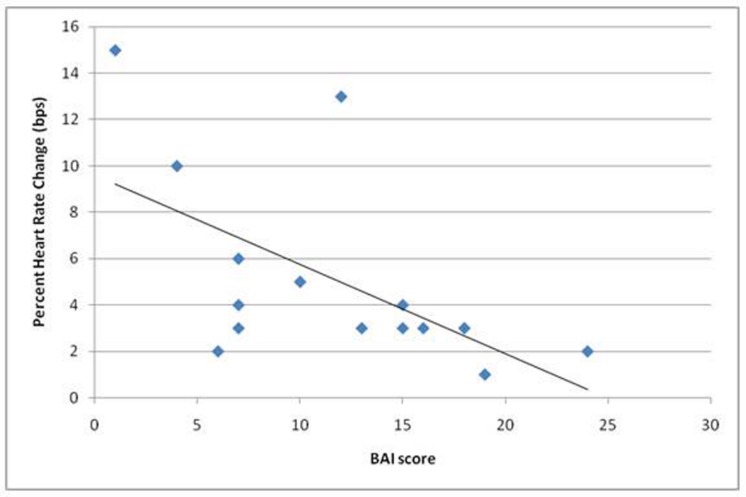
**Shows a significant negative correlation (*r* = -0.58) in performance on the task (given as average percent heart rate change across objective conditions when in the correct direction) and scores on the Beck Anxiety Inventory (BAI) scale (completed on the day of scanning)**.

Participants’ ability to volitionally regulate their heart rate, measured by percentage heart rate change in the intended direction (prompted increase or decrease) was negatively correlated with anxiety scores on the BAI, *r* = -0.58, *p* < 0.05. This suggests that participants who were less able to regulate their heart rate during this experiment experienced more anxiety symptoms (**Figure 4**).

### Neuroimaging Results

#### Main Effect of Objective (Cardiovascular Relaxation/Arousal) on Brain Activity

**FIGURE 5:**
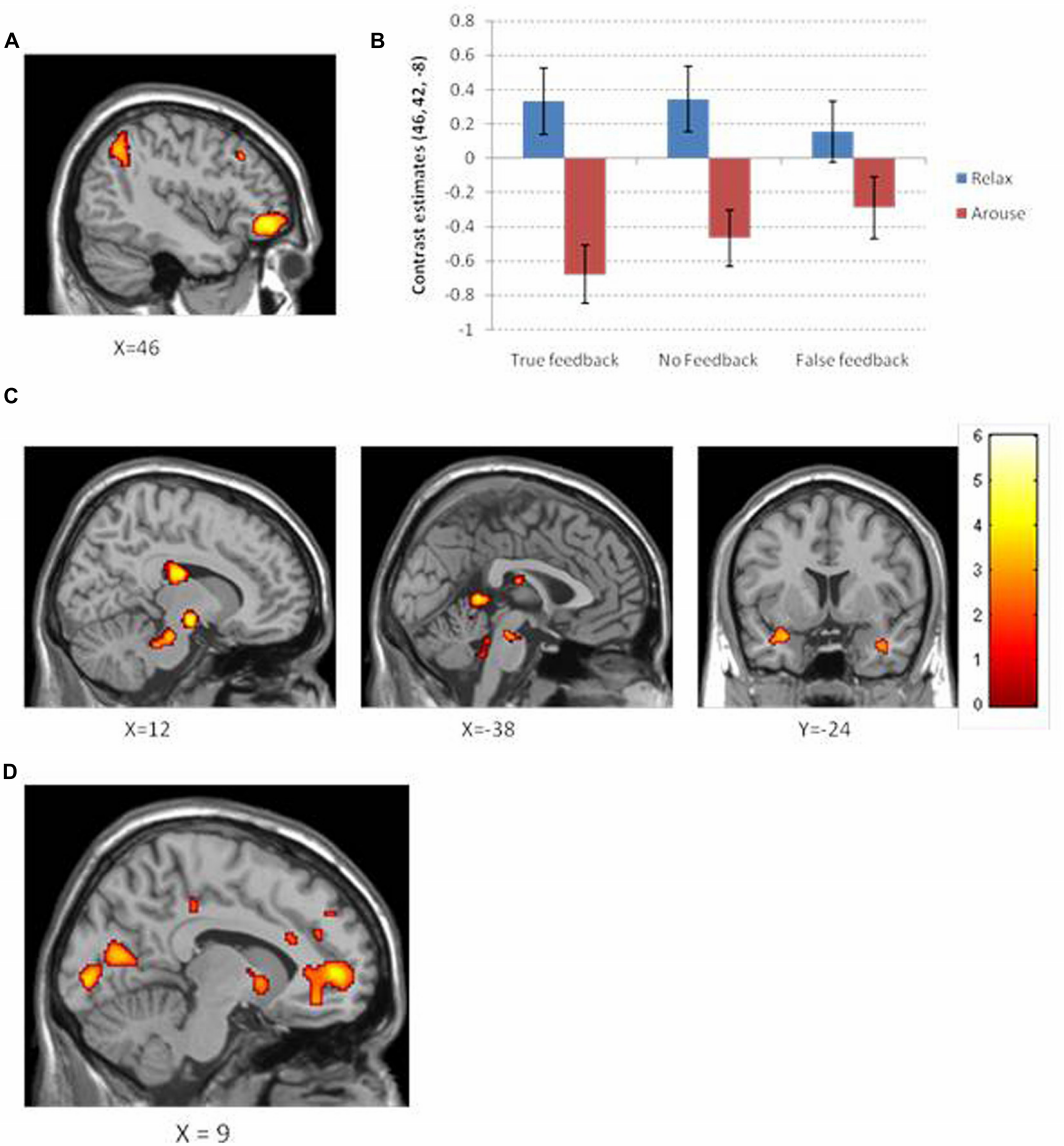
**(A)** Neural activation for the contrast relaxation > arousal observed in the right ventrolateral prefrontal cortex and right intraparietal lobule. **(B)** A plot of the contrast estimates at peak ventrolateral prefrontal voxel for objective and feedback type. Interestingly, the difference in relaxation and arousal for each feedback condition appears to mirror that observed in the behavioral data. **(C)** Neural activation for the contrast arousal > relaxation across different brain slices shows increased activation in the caudate, midbrain, and the insula/amygdaloid complex. Color bar corresponds to color maps on brain images which reflect the t statistic. **(D)** Neural activation which significantly correlates with ability to perform the task objective as measured by average percent change in heart rate. Increased activation shown for regions close to the midline within pregenual anterior cingulate, ventral striatum, and primary visual cortex.

The main effect on brain activity of intending to decrease heart rate was assessed by comparing relaxation and arousal tasks. Clusters of increased activity were observed in the right ventrolateral prefrontal cortex) and in the right inferior parietal lobule (see **Figure 5A**). Conversely, the main effect of intending to increase heart rate was assessed by conducting the reverse contrast, comparing arousal against relaxation conditions: this revealed greater activity within the left caudate, left midbrain, left posterior central gyrus, left cerebellar vermis, and a cluster encompassing regions of right amygdala and anterior insula (**Table 1**).

**Table 1:** Regional activity associated with main effect of task objective: decreasing heart rate (relaxation) versus increasing heart rate (arousal).

Region	Side	Coordinates of peak activity (MNI)	Voxels	Peak *T* score
**(A) Regional brain activity associated with intended heart rate decrease**
Ventrolateral PFC	Right	46 42 –8	323	5.50
Inferior parietal lobule	Right	50 –56 48	355	5.15
**(B) Regional activity associated with intended heart rate increase**
Caudate	Left	–16 –28 26	366	4.97
Midbrain	Left	–10 –10 –12	99	4.96
Cerebellar vermis		0 46 2	189	4.91
Amygdala, /insula	Right	32 10 –22	172	4.42

#### Main Effect of Feedback Type on Brain Activity

We first tested for a main effect of receiving veridical biofeedback across relaxation and arousal conditions (True > None + False). The presence of true biofeedback was associated with enhanced activation within the right occipito-temporal gyrus (Brodmann area 37). To ascertain whether this activation reflected biofeedback *per se*, or if it was driven by a visual representation of the moving thermometer, we performed separate contrasts of interest (**Table 2**). Significant activity was observed in the same region of the occipital-temporal gyrus for both true feedback and false feedback when they were compared to no feedback. However, this region was not activated when comparing true feedback to false feedback and vice versa. This suggests that this activation was primarily concerned with visual aspects of the feedback i.e., the moving bar. Blocks in which no feedback was received were associated with activity in left posterior cingulate and left anterior cingulate (*p* < 0.05 corrected) but interestingly only in the intended arousal condition (the same effect was not seen for relaxation).

**Table 2:** Regional activity associated with main effects of feedback type (True/None/False).

Region	Side	Coordinates of peak activity (MNI)	Voxels	T score
**(A) Regional brain activity associated with biofeedback (true feedback)**
*True > None*				
Occipito-temporal gyrus	Right	50 –66 –2	544	5.81
*True > False*				
NSA				
**(B) Regional activity associated with no feedback**
*None > True*				
Posterior insula	Right	38 –18 18	40	3.51*
*None > False*				
NSA				
**(C) Regional activity associated with false feedback**
*False > None*				
Occipito- temporal gyrus	Right	46 –66 2	740	5.87
*******False > True*				
NSA		

NSA, no suprathreshold activity. Clusters reported at p < 0.05 corrected. *SVC, small volume corrected.

#### Modulation of Brain Activity Related to Objective (Cardiovascular Relaxation/Arousal) By Feedback Type

By examining the interaction between feedback and objective, we attempted to identify regions where heart rate relaxation/arousal related activity was modulated by the type of feedback. There were no clusters of activity reflecting this interaction at a FWE correction (or at a more permissive uncorrected threshold of *p* < 0.001). To obtain an impression of how feedback type may have differentially activated brain regions involved in relaxation and arousal, individual *t*-tests were performed to assess the effects of intended relaxation and intended arousal under each feedback condition (**Table 3**; **Figure 5**). In these tests, of note are the observations first; that the absence of feedback during the intended arousal conditions evoked greater engagement of right insula/amygdaloid complex, and second; that the presentation of false or no feedback during the intended relaxation condition enhanced activation within regions of subgenual cingulate cortex.

**Table 3:** *Post hoc* tests showing regions of activation associated with feedback × objective.

Region	Side	Coordinates of peak activity (MNI)	Voxels	T score
**(1) True Feedback**
*(A) Heart Rate Increase > Decrease (relaxation > arousal)*
Superior temporal gyrus	Right	62 –46 –4	136	4.93
Intraparietal sulcus*	Right	32 –66 48	318	4.23
vlPFC	Right	46 42 –8	117	3.91
*(B) Increase > Decrease*
Thalamus	Right	2 –16 16	74	4.13
**(2) No Feedback**
*(A) Decrease > Increase*
Orbitofrontal gyrus/anterior insula*	Right	22 28 –8	37	4.68
*(B) Increase > Decrease*
Insula/amygdala*	Right	32 10 –24	72	4.39
**(3) False Feedback**
*(A) Decrease > Increase*
Subgenual anterior cingulate	Left	–12 36 –2	170	4.71
*(B) Increase > Decrease*
NSA	NSA	NSA	NSA	NSA

*Nominal description of region refers to anatomical extent of cluster which may encompass more than one region, the coordinates of the peak voxel represent the location maxima of the cluster. NSA, no suprathreshold activity.

#### Brain Activity Related to the Magnitude of Successful Task Performance

We performed a group-level analysis to test for regional activity correlating with successful task performance, defined as the average percent change in heart rate across objective conditions. Regional activity covarying with performance was observed within pregenual anterior cingulate, angular gyrus, middle temporal gyrus, temporal pole, ventral striatum, and primary visual cortex (calcarine cortices; *p* < 0.05 corrected; **Figure 5D**).

#### Brain Activity Mapping Heart Rate and Breathing Rate Across Experimental Conditions

We further tested for regional brain activity related to changes in heart rate (the primary task objective) and also breathing rate during each scan. These physiological variables formed the two single regressors within the same analytic design matrix. Breathing rate over the course of the experiment was associated with significant changes within the right insula. This is unlikely to reflect a consequence of the instruction to try to maintain a constant breathing rate across increased and decreased heart rate blocks, since task conditions were implicitly controlled for by inclusion of heart rate within the same regression analysis. At an uncorrected threshold only (*p* < 0.001), heart rate changes correlated with activity within periaqueductal gray matter, right caudate nucleus, and right insula cortex.

## Discussion

The central regulation of internal bodily states is crucial to adaptive behavior, and controlled proximately through autonomic nervous system and viscerosensory afferents. Most psychological models for understanding the interaction between mind and body underplay organ specificity and patterning across peripheral responses (Harrison et al., 2010). Nevertheless, emotional and motivational feelings are linked to the prediction and signaling of physiological ‘interoceptive’ state (Seth, 2013; Seth and Critchley, 2013). Thus, by studying brain mechanisms controlling autonomic reactivity, specifically those underlying the generation and feedback representation of changes in internal state, a more comprehensive integrated neurobiological account of affective behavior can be achieved.

The present study illustrated neural mechanisms associated with the volitional modulation of heart rate. Individual task performance varied across participants; even so, the aim of eliciting increases and decreases in heart rate for intended arousal and intended relaxation, respectively, was achieved by all but one of the participants. Most strikingly, the activity within ventrolateral prefrontal cortex (Brodmann areas 44, 45, and 47; with peak activation in the lateral orbitofrontal/inferior frontal gyrus or Brodmann area 47) was enhanced during ‘active relaxation’ conditions intended to decrease heart rate. This broad region is implicated in the cognitive appraisal of emotional events and corresponding behavioral control (to meet task demands; Lee and Siegle, 2012). Furthermore, ventrolateral prefrontal cortex receives motivational and emotional information from the orbitofrontal cortex and subcortical areas (midbrain, hypothalamus, and striatum) and, in non-human primates, supports the computation of the behavioral significance of external events for goal directed behavior (Sakagami and Pan, 2007). Ventrolateral prefrontal cortex is likely to influence autonomic function indirectly through influences on a network incorporating visceral cingulate and insular cortices alongside amygdala and dorsal brainstem. Previous neuroimaging studies of emotion regulation also implicate this ventrolateral prefrontal region in the volitional control of physiological arousal (Beauregard et al., 2001; Critchley et al., 2002a), and during the voluntary suppression of negative affect during cognitive reappraisal (Phan et al., 2005). This prefrontal region is also involved in evaluating and gating the influence of contextual emotional information in decision-making (Beer et al., 2006); for example, it is engaged when decisions are made in states of high, but not low, urgency, suggesting that it may suppress anxiety and emotional arousal associated with risky decision-making (Jones et al., 2011). The present study extends these data by indicating that ventrolateral prefrontal cortex has a steering role in the intentional regulation of bodily arousal.

Activation within right inferior parietal lobule was also enhanced during intentional relaxation/heart rate reduction. This region is implicated in directed attention toward external stimuli (Fink et al., 1996), and earlier data suggest a shared neural substrate for selective attention and autonomic arousal (Critchley et al., 2000b). Interestingly in the present study, this region was engaged during relaxation conditions particularly when receiving veridical biofeedback, consistent with its potential role as a substrate for body-centered integration of external feedback signals with internal arousal state. Conversely, the intention to increase heart rate through enhancing one’s state of arousal activated regions within the amygdala, midbrain, and caudate. Amygdala activation is linked to the generation of transient sympathetic (electrodermal) response (Phelps et al., 2001; Williams et al., 2001) and suppression of the baroreflex, allowing blood pressure and heart rate to rise together (Gianaros et al., 2012). The amygdala contributes to a network of regions including anterior cingulate cortex, insula and periaqueductal grey matter (PAG), which mediates cardiovascular reactions to psychological stressors (Gianaros et al., 2004, 2012; Gray et al., 2009; Wager et al., 2009). Additionally, both caudate and midbrain are implicated in autonomic nervous system regulation and dysregulation (e.g., Beacher et al., 2009; Gray et al., 2009). The present study adds to this literature by highlighting the capacity for individuals to engage volitionally this set of subcortical brain regions. While intentional behavioral responses are typically thought to originate from processes within prefrontal cortex, the present study suggests that intentional changes in autonomic arousal state may also be engendered through more direct recruitment of a select network of subcortical structures linked to motivational behavior.

Across task conditions, we showed an interesting relationship between neural activity and the participants’ success at performing the instructed directional change in heart rate. This success was quantified as the magnitude of increase in heart rate during the intended increase/arousal conditions and the magnitude of decrease in heart rate during the intended decrease/relaxation conditions. Successful performance was associated with activation across regions including pregenual anterior cingulate, ventral striatum, and early visual cortex (illustrated **Figure 5D**) and lateral parietal and temporal cortices and temporal pole. Interestingly, earlier neuroimaging studies of biofeedback, with sympathetic electrodermal signals, implicate similar a pregenual cingulate response to task success alongside amygdala/rostral temporal lobe that also predicting the rate of (successful) physiological relaxation (Critchley et al., 2001). Our present findings includes the ventral striatum in these processes, suggesting the presence of a reward (prediction error) signal that bridges the cognitive intention to perform the task linked to the monitoring of physiological change. The fact that these findings occurred independently of perturbation in visual feedback also suggests that anterior cingulate, ventral striatal, and temporal regions are coupled to internalized interoceptive information.

We observed no formal interaction between visual feedback and task objective. It was not predicted that the experimental feedback manipulations would have little impact on task performance or associated brain activity. The main feedback-related observations were of visual cortex activation by true and false visual feedback, and of enhanced activity within posterior insula in the absence of feedback. The latter observation is in keeping with the notion of greater attention-driven engagement of interoceptive process mediated by insula cortex in the absence of veridical feedback, although previous studies also present this argument for increases in insula activity evoked by false feedback (Critchley et al., 2002a). Overall, the volitional control of heart rate seemed to be a concept that all participants could grasp and attain from minimal practice with veridical feedback before the scanning session. However, in the scanner, the feedback appeared to provide little additional value for the participants to achieve intended changes in physiological state. Interestingly, this seems to suggest that heart signals (in contrast to other internal autonomic responses such as electrodermal activity) are more readily accessible for volitional control, at least for those who score lower on anxiety measures. Retrospectively, it is an omission that we did not explore this further by measuring individual differences in interoceptive ability using heart rate detection tasks (Garfinkel et al., 2015). There was some indication from the planned simple contrasts (presented in **Table 3**) that the quality of visual feedback modulated the neuro cognitive strategies employed to reach the target volitional state of increased or decreased heart rate. This is consistent with the observation that, with extensive training, people can become more adept at using biofeedback as a means to regulate efficiently their arousal state (Nagai et al., 2004b). Ultimately, the biofeedback manipulation only partially addressed a secondary question of this study regarding mechanisms for volitional manipulation of heart rate and their neural correlates.

One hypothesis was that ‘viscerosensory’ insular cortex, including anterior insula, would contribute to the neural circuitry supporting the volitional regulation of heart rate, by virtue of its evident role in the integration of cognitive, exteroceptive, and interoceptive information, and its relationship to forebrain visceromotor regions including anterior cingulate and amygdala: human insula is implicated in autonomic control, interoceptive representations, and emotional feelings (Penfield and Faulk, 1955; Oppenheimer et al., 1992; Craig, 2002; Critchley et al., 2004; Meyer et al., 2004; Harrison et al., 2010; Jones et al., 2010). Yet it is also clear that the insula does not act in isolation, neither in its contribution to autonomic regulation, nor as a substrate for feelings states and interoception. Anterior insula, along with anterior cingulate and amygdala, is implicated in ‘translating’ interoceptive bodily signals into feeling states (Craig, 2002, 2009; Critchley et al., 2004; Gray et al., 2007; Singer et al., 2009). It is also implicated as one cortical hub within a network for salience and self- representation (Seeley et al., 2007; Sridharan et al., 2008) and connects with frontotemporal hubs that contribute to contextual social as well as emotional behavior (Ibanez and Manes, 2012; Limongi et al., 2014). In these scenarios, anterior insula is prosed to serve as a comparator within the more distributed predictive coding of emotion, putatively receiving efference copies of descending signals from anterior cingulate and prefrontal cortices (Critchley, 2005; Paulus and Stein, 2006; Singer et al., 2009; Critchley and Seth, 2012; Seth, 2013). It is therefore noteworthy that in the present study, we did not observe marked engagement of anterior insular cortex across the different task conditions. Increased anterior insula engagement when decreasing heart rate in the absence of a feedback signal is present, but its interpretation tempered by the absence of an overarching interaction. The most parsimonious account is that we showed little behavioral or neural evidence for the integration of exteroceptive feedback information with interocetive processes toward successful task performance. Task-related increases in heart rate/arousal preferentially engaged ventrolateral prefrontal cortex, rather than anterior insula. While activity related to successful task performance evoked change within insula, including a region of anterior insula, even this effect was attenuated relative to the activity observed the pregenual cingulate, ventral striatum and even primary visual cortex. In earlier biofeedback studies of electrodermal activity, anterior insula engagement is typically associated with interference caused by perturbed feedback (e.g., Critchley et al., 2002a) and there was weak evidence showing a similar effect in the present study.

We anticipated that scan-by-scan fluctuation in measured physiological indices, i.e., heart rate and respiration, would be reflected in changes in neural activity within posterior insula (implicated as primary interoceptive cortex). We did observe activity within right posterior insula related to increased respiration and a weaker positive correlation with heart rate change. However, there was a stronger correlation between heart rate and activity within both right caudate nucleus and midbrain (PAG). The integrity of the caudate is linked to autonomic response tendencies (Beacher et al., 2009). Caudate activity, alongside insula and dorsal cingulate, predicts heart rate changes to emotional stimuli (Critchley et al., 2005) and at a network level, the connectivity between caudate, cingulate, insula, and midbrain is coupled to resting state fluctuations in heart rate variability (Chang et al., 2013). Together these data suggest a proximate network of brain regions supports the representation of heart rate signals, which are selectively engaged in their volitional control. These inferences merit further investigation. Different methodological approaches may shed more light: for example, we focused on heart rate change (measured with pulse oximetry) as an intuitively accessible interoceptive response: however, potentially more accurate methods for mapping the central control of heart rate involve measuring heart rate variability, reflected in changing intervals between successive heartbeats (on electrocardiography: R–R intervals). Heart rate variability reflects homeostatic regulation through sympathetic and parasympathetic axes of the autonomic nervous system, where high frequency components index vagus-mediated coupling of cardiac control with respiration (Napadow et al., 2008). Somewhat impressively, participants were able to induce significant changes in their heart rate while maintaining a consistent breathing rate, suggesting cognitive mechanisms are sufficient to fulfill the task objectives, bypassing the need to evoke cardiorespiratory reflexes through explicitly modulating the rate or depth of breathing. Typically, humans regulate their breathing in order to become more relaxed, e.g., in yoga or meditation. In emotional situations involving high physiological arousal, breathing increases to provide muscles with more oxygen as part of the fight or flight response. Such effects did not account for task-associated changes in heart rate in the present study. Nevertheless, while participants were asked to maintain a constant breathing rate, their breathing fluctuated across the course of the experiment and was tracked by activity within right insular cortex. This finding is consistent with evidence from studies in humans and other animals that map reciprocal respiratory projections between insula and vagus nerve (Radna and MacLean, 1981) and which show strong inhibitory effects of insula stimulation on respiration rate (Kaada and Jasper, 1952; Hoffman and Rasmussen, 1953).

Interestingly, there was a significant negative correlation between the ability of participants to modulate their heart rate intentionally and anxiety scores. Those who were rated as more anxious were less able to meet the task objective (i.e., increase or decrease their heart rate). Our findings suggest that greater anxiety is associated with impaired capacity for physiological control and, by implication, a relatively reduced ability to contain emotional arousal responses as effectively as their less-anxious individuals. This observation in a subclinical group qualifies evidence showing that individuals with clinical anxiety show heightened sensitivity to interoceptive cues (Domschke et al., 2011) and associating autonomic dysregulation with anxiety disorders (Wilhelm et al., 2001). Self-regulation of autonomic arousal may be applied as every day countermeasures or in therapeutic interventions to enhance the regulation of emotions states, notably anxiety. The contribution of bodily arousal states is well-recognized, highlighting a link between interoceptive processes to anxiety symptoms (Clark, 1986). However, the simplified hypothesis that individual differences in (heightened) interoceptive sensitivity, quantified for example by assessing an individual’s accuracy in detecting own heartbeats at rest, predisposes to anxiety has limited validity in both normative and clinical populations. Overrepresentation of individuals with enhanced interoceptive processing (heartbeat detection) is observed in populations with anxiety (Pollatos et al., 2007; Dunn et al., 2010; Terasawa et al., 2013), although the finding is not always demonstrated (Asmundson et al., 1993; Craske et al., 2001). This inconsistency reflects a complexity addressed by the theoretical proposal, backed by data, of dissociable cognitive dimensions to interoception wherein awareness and subjective perception/interpretation of bodily response may diverge significantly from objective measures of interoceptive sensitivity accuracy (Garfinkel and Critchley, 2013; Garfinkel et al., 2015). Discrepancy between these subjective (prediction/interpretation) and objective (interoceptive accuracy) dimensions is proposed to give rise to emotional symptoms through ‘interoceptive prediction error’ signaling an impaired sense of control of internal physiological state (Paulus and Stein, 2006; Seth, 2013). Our finding, in a subclinical group, that better volitional control of heart rate predicts lower levels of state anxiety is consistent with this concept. Equally, the counter-argument that anxiety impairs non-specifically performance of volitional control tasks must acknowledge the bidirectional psychophysiological dynamics of symptom expression.

Human anxiety consists of a complex pattern of cognitive, affective, physiological, and behavioral changes in response to threat, loss, or perceived negative outcome (Beck and Clark, 1997). The finding that individuals with greater anxiety are significantly less able to volitionally modulate their heart rate without prior training has clinical implications for treatment approaches. Although, the presence of biofeedback did not significantly improve participant’s ability to regulate their heart rate on this one occasion, heart rate feedback retains potential therapeutic utility for anxiety patients. Visual heart rate feedback is reported to facilitate exposure treatment of animal phobic patients (Nunes and Marks, 1975) and auditory heart rate feedback enhances claustrophobia treatment (Telch et al., 2000). Conversely, increased anxiety can be induced by false heart rate feedback in patients with panic disorder (Ehlers et al., 1988). Thus, heart rate based biofeedback paradigms have the potential to enhance ‘interoceptive exposure’ in the management of anxiety disorders. There is evidence to support the notion that autonomic biofeedback training may also diminish symptoms in other patient groups with stress-sensitive neuropsychiatric and medical disorders, including epilepsy (Nagai et al., 2004a; Micoulaud-Franchi et al., 2014), tic disorder (Nagai et al., 2009) and cardiovascular disease (Moravec and McKee, 2011). There is therefore broader utility of biofeedback approaches in managing dissociative neuropsychiatric symptoms (Sedeño et al., 2014).

There are limitations to this study: training participants in performing biofeedback prior to scanning and ensuing all could carry out biofeedback to a reasonable standard may have reduced participant variability in task performance and increased the chance of observing feedback-specific influences. Also, the instructions for participants to increase or decrease their level of arousal may have biased them toward engaging mechanisms that go beyond those necessary purely for the volitional regulation of heart rate. If this were the case, the findings we observed within the brain (related to the task intention and correlating with task achievement) may reflect other psychological processes (e.g., mediating wakefulness or emotionality) that are incidental to, though not independent of, the participants’ directed regulation of their physiological arousal. However, all participants were aware that they were only required to increase or decreased their heart rate in accordance with task instructions. Moreover from the outset we defined the nebulous terms arousal and relaxation to refer operationally to cardiovascular arousal and relaxation (i.e., increased and decreased heart rate). No instructions were given to change level of alertness, wakefulness, or direct attention to emotional events. Our study suggests that the volitional regulation of cardiovascular arousal, at least within the setting of a neuroimaging experiment, is relatively easy to attain with minimal practice and no need for active feedback. While we extrapolate our findings to suggest that these same brain regions associated with task success may be engaged in similar mechanisms to regulate physiological arousal contributing to anxiety states, this proposal will require direct empirical validation. At a technical level, coverage of ventromedial prefrontal cortex during the acquisition of echo planar T2^∗^ datasets was not always consistent across participants, diminishing our ability to infer the contribution of this region to the regulation of autonomic state: previous studies report inverse correlations between ventromedial/orbitofrontal cortex activity and sympathetic arousal (Nagai et al., 2004b).

To summarize, our data provide evidence for the role of specific brain regions, notably ventrolateral prefrontal cortex, in the volitional control of heart rate, with implications for understanding, and treating anxiety and stress-sensitive neuropsychiatric and physical conditions. These regions are linked to wider functional brain networks implicated in emotional regulation. Interestingly we did not provide strong evidence for our prediction that insula cortex was critical to the volitional regulation of heart rate through biofeedback. However, our participants were relatively naïve to the use of biofeedback techniques and their overall task performance was not shaped by the presence of veridical visual feedback, but reflects the employment of alternative strategies to implement the directed task objectives. Nevertheless, we highlight the cortical and subcortical networks mediating intentional autonomic cardiac control. Understanding these mechanisms has implications for management of clinical disorders of emotion regulation, and relevance to training self-management using biofeedback approaches, including neurofeedback with fMRI.

## Conflict of Interest Statement

The authors declare that the research was conducted in the absence of any commercial or financial relationships that could be construed as a potential conflict of interest.
